# *In Vitro* Differentiation of First Trimester Human Umbilical Cord Perivascular Cells into Contracting Cardiomyocyte-Like Cells

**DOI:** 10.1155/2016/7513252

**Published:** 2016-03-30

**Authors:** Peter Szaraz, Matthew Librach, Leila Maghen, Farwah Iqbal, Tanya A. Barretto, Shlomit Kenigsberg, Andrée Gauthier-Fisher, Clifford L. Librach

**Affiliations:** ^1^Create Fertility Centre, Toronto, ON, Canada M5G 1N8; ^2^Institute of Medical Sciences, University of Toronto, Toronto, ON, Canada M5S 1A8; ^3^Department of Obstetrics and Gynecology, University of Toronto, Toronto, ON, Canada M5G 1E2; ^4^Department of Physiology, University of Toronto, Toronto, ON, Canada M5S 1A8; ^5^Department of Obstetrics and Gynecology, Women's College Hospital, Toronto, ON, Canada M5S 1B2

## Abstract

Myocardial infarction (MI) causes an extensive loss of heart muscle cells and leads to congestive heart disease (CAD), the leading cause of mortality and morbidity worldwide. Mesenchymal stromal cell- (MSC-) based cell therapy is a promising option to replace invasive interventions. However the optimal cell type providing significant cardiac regeneration after MI is yet to be found. The aim of our study was to investigate the cardiomyogenic differentiation potential of first trimester human umbilical cord perivascular cells (FTM HUCPVCs), a novel, young source of immunoprivileged mesenchymal stromal cells. Based on the expression of cardiomyocyte markers (cTnT, MYH6, SIRPA, and CX43) FTM and term HUCPVCs achieved significantly increased cardiomyogenic differentiation compared to bone marrow MSCs, while their immunogenicity remained significantly lower as indicated by HLA-A and HLA-G expression and susceptibility to T cell mediated cytotoxicity. When applying aggregate-based differentiation, FTM HUCPVCs showed increased aggregate formation potential and generated contracting cells within 1 week of coculture, making them the first MSC type with this ability. Our results indicate that young FTM HUCPVCs have superior cardiomyogenic potential coupled with beneficial immunogenic properties when compared to MSCs of older tissue sources, suggesting that* in vitro* predifferentiation could be a potential strategy to increase their effectiveness* in vivo*.

## 1. Introduction

Following an acute myocardial infarction (MI), excessive cardiomyocyte loss is associated with a cascade of events resulting in ventricular decompensation and congestive heart failure (CHF) [1]. Despite advances in pharmacological, interventional, and surgical therapies, CHF contributes to significant morbidity and mortality worldwide. The heart is a partially self-renewing organ in which stem cells play an important restorative role: both endogenous bone marrow-derived and heart tissue-derived stem cells participate in cardiac regeneration following ischemic injury [2–4]. However, in aged patients, this regenerative capacity is diminished and generally insufficient. The implantation of healthy donor cells into the damaged myocardium to replace lost cardiomyocytes and restore ventricular structure and function has been investigated since the 1990s. Various groups reported the facilitation of remodeling and enhancement of inherent healing mechanisms in preclinical models of myocardial infarction (MI) [3–6]. The regenerative effects of the implanted cell candidate are expected to be mediated at least in part through the emergence of new cardiomyocytes at the injury site.

The successful differentiation of stem cells into cardiomyocytes is defined via the expression of cardiac lineage markers. The elevation of early differentiation markers, often transcription factors (NKX2.5, Mef2C, and GATA4) [7, 8], can indicate the successful initiation of the cardiomyogenic process. Mature cardiomyocyte markers (SIRPA) [9], in particular those with cardiomyocyte-specific structure and function (cardiac troponin T, cTnT) [10], heavy chain cardiac myosin MYH6 [7, 11, 12], or those required for the coordinated function of cardiac muscle (connexin 43) [13–15], are hallmarks of the terminal cardiomyocyte phenotype. In tissue engineering the abovementioned markers are routinely used to assess differentiation efficacy [9, 16].

In order to achieve the highest cardiomyogenic differentiation efficiency and purity, the differentiation of embryonic stem (ES) cells and pluripotent stem (PS) cells into functional cardiomyocytes has been thoroughly described and optimized at the level of inductive factors and their exact timing of action [16–20]. These strictly controlled* in vitro* differentiation methods gave rise to multiple cardiac engineering projects worldwide (EHM) [21–24]. However treating patients with ESC- or iPSC-based engineered tissues still poses unsolved challenges, including suboptimal immunological and electrophysiological features [25, 26] as well as ethical and safety controversion.

Since their description in 1995, mesenchymal stromal cells (MSCs), in particular bone marrow MSCs (BMSCs), have gained substantial interest in tissue regeneration including heart repair [27]. According to the most recent consensus, MSCs are resident regenerative cells that can be found virtually in any vascularised tissue [28]. Once isolated, MSCs are easy to expand and culture, and they possess immunomodulatory and immunoprivileged properties and have extensive paracrine effects [29, 30]. Several preclinical studies have demonstrated their safety and efficacy in cardiac regeneration [3, 6].

MSC differentiation strategies have thus far involved the use of pharmacological agents such as 5-azacytidine [27] and DMSO [31] and growth and morphogenic factors like BMP-2 [32, 33] or angiotensin-II [34]. Harder to define and control, yet physiologically more relevant strategies were based on the hypothesis that the cardiac microenvironment itself can grant the multifaceted inductive effect MSCs may need for cardiomyogenic transformation and commitment. The application of cardiac cell lysates on BMSC cultures [35], direct coculture of MSCs with cardiomyocytes* in vitro* [14, 36], or the implantation of MSCs into the ventricular myocardium of small animals* in vivo* [37, 38] resulted in increased cardiac marker expression in MSCs. Previous reports suggested that cardiomyogenesis occurred spontaneously after treating cardiac injuries with MSCs [39–42], although* de novo* cardiomyocytes also arose from the fusion of BMSCs and cardiomyocytes [43, 44]. Earlier several groups reported the generation of functional, spontaneously contracting cardiomyocytes from non-human MSC-like cells [31, 45] but to our knowledge the derivation of functional cardiomyocytes from human MSCs of any tissue source has not been reported.

The perivascular region of human umbilical cord tissue is a rich source of MSCs with pericyte properties [46–48]. The cardiomyogenic potential and advantages of human umbilical cord perivascular cells (HUCPVCs) over BMSCs have been demonstrated by several groups, both* in vitro* [46, 49] and* in vivo* [50–52]. We have previously shown that, in comparison with term HUCPVCs, FTM HUCPVCs have increased proliferative capacity and higher multilineage differentiation capacity, including the cardiomyogenic lineage [46]. We hypothesized that young, multipotent FTM HUCPVCs have greater overall cardiomyogenic potential when compared to term HUCPVCs or BMSCs. In order to further investigate and compare the cardiomyogenic differentiation potential of HUCPVCs and BMSCs, we employed single cell suspension and aggregate-based direct cocultures of HUCPVCs and BMSCs with primary cardiomyocyte feeder layers to induce their differentiation into functional heart muscle-like cells.

## 2. Methods

### 2.1. Tissue Culture and Cell Labeling

All studies were performed with institutional research ethics board approval (REB number 454-2011, Sunnybrook Research Institute; REB 29889, University of Toronto, Toronto, Canada). Previously established lines of FTM HUCPVCs and term HUCPVCs (*n* ≥ 3 independent lines for each) [47] and a commercially available line of bone marrow MSCs (Lonza) were cultured in alpha-MEM (Gibco) supplemented with 10% FBS (Hyclone) and penicillin/streptomycin cocktail (Gibco) and passaged at 70–80% confluency. Rodent primary cardiomyocyte cultures were kept in DMEM-F12 containing 10% FBS (Hyclone) and penicillin/streptomycin cocktail (Gibco). MSC, monocyte cocultures were kept in RPMI supplemented with 10% FBS (Hyclone) and penicillin/streptomycin. Cell cultures were kept in humidified incubators (37°C, 5% CO_2_).

All animal procedures were approved by the Animal Care Committee of the University Health Network (Toronto, Canada) and all animals received humane care in compliance with the* Guide for the Care and Use of Laboratory Animals* (National Institutes of Health 1996). As described previously [46], primary rat cardiomyocyte cultures were prepared from rats sacrificed at p2-5. Heart ventricles were minced and agitated at 37°C in 1.5% trypsin PBS solution. Primary cultures were treated with BrdU (16 h, 5 *μ*M) and washed prior to addition of MSCs 24 h later. Dissociated MSCs were added to cardiomyocyte monolayers to achieve 5% MSC content in each well. Alternatively MSCs were labeled with viable, nontransferable fluorescent dye (CellTracker Green, Invitrogen 5 *μ*M, 1 h) prior to transfer, in order to visualize integrating MSCs.

Aggregate formation was induced by hanging 25 *μ*L drops of cell culture media containing 500 MSCs each (passage# ≤ 6). After 3 days, aggregates were transferred onto primary rat cardiomyocyte monolayers and kept in coculture for over 7 days. Alternatively aggregates were incubated with viable, nontransferable fluorescent dye (CellTracker Green, 5 *μ*M, 1 h) prior to transfer, in order to better visualize and record developing aggregates. Aggregate cocultures were dissociated (trypsin 0.25%, 3 min) for flow cytometry (FC) or fluorescence-activated cell sorting (FACS) analysis.

ReproCardio*™* human induced pluripotent stem (hiPS) cell derived cardiomyocytes (ReproCell, Japan) were used as positive control for cardiomyocyte marker gene and protein expression analysis.

### 2.2. Flow Cytometry and FACS

For FC and FACS, cell cultures were dissociated with 0.25% trypsin/EDTA solution (3 min, 37°C). Cell suspensions were incubated with fluorophore conjugated primary antibodies according to providers description (1 : 40, 30 min 4°C). FC analysis was performed using either analogue (FACSCalibur, BD; Create Fertility Centre, Toronto) or digital (LSR II, Canto II, BD; UHN SickKids Flow Cytometry Facility, Toronto) analytical cytometers. FACS was performed using digital cell sorters (MoFlo Astrios, Aria II, UHN SickKids Flow Cytometry Facility, Toronto) and sorted cells were replated within 1 hour after the procedure. For gating strategy of TRA-1-85 human cell surface antigen, see Supplementary Figure 1A in Supplementary Material available online at http://dx.doi.org/10.1155/2016/7513252. Antibodies applied for FC were as follows: anti-TRA-1-85 FITC, anti-TRA-1-85 APC and anti-connexin 43 APC (R&D Systems), anti-HLA-G FITC, anti-HLA-A FITC, anti-SIRPA FITC (AbD Serotec), anti-SSEA4 FITC, anti-CD146 FITC (BD Biosciences), and anti-CD49f PE (BioLegend). Fluorescent signals were gated to isotype controls matching the applied antibodies and fluorophores.

### 2.3. Immunocytochemistry and Microscopy

Cells grown on chamber slides (BD) were fixed (paraformaldehyde 4% in PBS, 15 min). Every following step was performed in PBS containing 1% BSA (Sigma). Cells were permeabilised (0.1% Triton X-100, 25 min) and blocked with 5% normal goat serum (NGS, 15 min). Primary antibody incubation was performed at 4°C overnight, dilution 1 : 200. Primary antibodies, anti-HuNu (Millipore), anti-cTnT (Abcam), anti-Mef2c (New England Biolabs), anti-connexin 43 (Abcam), and anti-alpha-sarcomeric actinin (Abcam), were washed and samples were incubated with secondary antibodies (1 : 500) for 30 minutes at RT. Secondary antibodies used were as follows: anti-rabbit-Alexa488, anti-mouse-Alexa594, and anti-rabbit-Alexa555 (Invitrogen) and anti-mouse Alexa555 (Cell Signaling).

Stained specimens were kept in mounting media (PermaFluor, ThermoScientific) and images were acquired using a fluorescent microscope (EVOS, Life Technologies) or spinning disk confocal microscope (Quorum Zeiss AxioVert, SickKids Imaging Facility, Toronto). Images were quantified using Volocity*™* (Perkin-Elmer) imaging software.

### 2.4. RNA Isolation and Quantitative RT-PCR

Undifferentiated hMSC cultures or hMSCs sorted from cocultures (TRA-1-85^high^) were lysed with RLT lysis buffer (Qiagen). RNA samples were prepared using the RNAeasy kit (Qiagen), according to the manufacturer's instructions. cDNA was prepared using RNA to cDNA EcoDry Premix (Clontech), according to the manufacturer's instructions. For MY6H analysis, Taqman qPCR primer assays were used (Applied Biosystems) with IDT PrimeTime qPCR probes. For cTnT analysis, primers were synthesized (Sigma) and the reaction mixture was prepared using the Rotor-Gene SYBR Green PCR kit (Qiagen). Human cardiac RNA positive control was from Ambion. qPCR was performed on the Rotor-gene 6000 instrument (Qiagen) using 10 ng of cDNA per reaction. Gene expression levels were determined by PCR Array Data Analysis Centre (SABiosciences) using the delta-delta Ct (ΔΔCt) method and reported as fold increase compared to undifferentiated cells. GAPDH was used as a normalizer. All assays were done in triplicate for at least 3 independent experiments. Oligo sequences are as follows: CTnT_F: GGC AGC GGA AGA GGA TGC TGA A; CTnT_R: GAG GCA CCA AGT TGG GCA TGA ACG A; MYH6_F: GCA AAG TAC TGG ATG ACA CGC T; MYH6_R: GTC ATT GCT GAA ACC GAG AAT G.

### 2.5. Monocyte Cytoxicity Assay

Human blood was obtained with written informed consent from healthy volunteers (REB number 28889, University of Toronto, Toronto, Canada). Mixed lymphocytes were isolated using Ficoll gradient centrifugation. Cell concentration, viability, and purity were determined by microscopy using trypan blue. Lymphocytes were incubated with MSC monolayers at a ratio of 10 : 1 for 72 h. Colorimetric LDH measurement (Roche) was performed using cell free medium after lymphocyte incubation and quantified with plate reader (FilterMax F5, Molecular Devices) at 490 nm.

## 3. Results

### 3.1. Human Umbilical Cord Perivascular Cells Have Increased* In Vitro* Cardiomyogenic Differentiation Potential Compared to BMSCs

**Figure 1:**
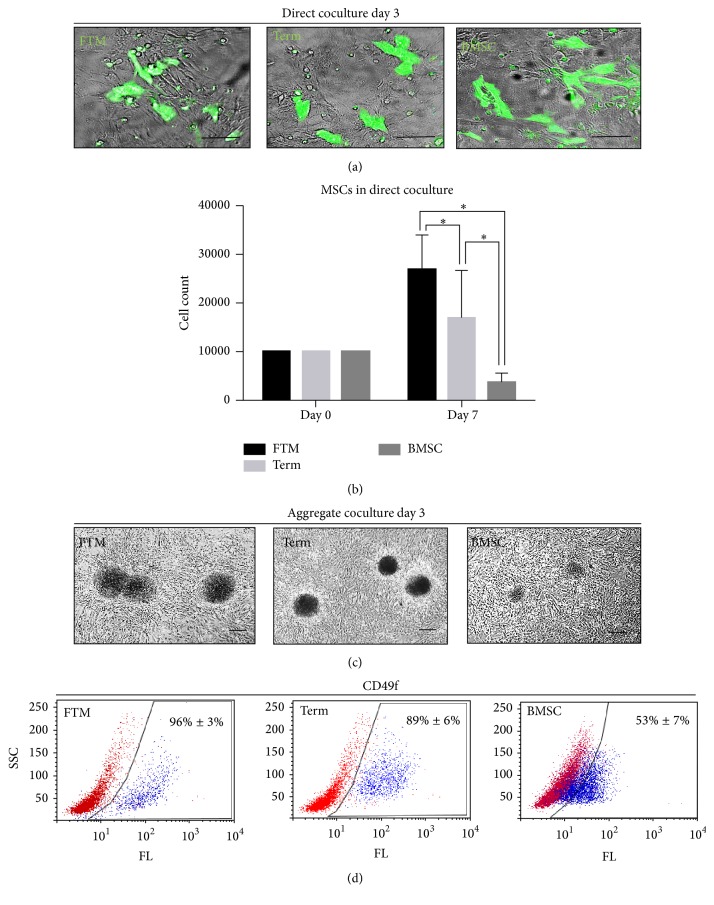
Cardiomyogenic induction of hMSCs using primary rat cardiomyocyte coculture strategies. (a) Bright field, fluorescent microscopy (green) overlay of direct cocultures from prestained human MSCs (CellTracker Green) and rat primary cardiomyocytes at day 3. Bar = 100 *μ*m. (b) Cell counts of human cells (TRA-1-85^high^) extracted from hMSC, rat cardiomyocyte cocultures at day 7, compared to initial cell counts (day 0). (c) Bright field microscopy images of hMSC aggregate cocultures. (d) Flow cytometry analysis: CD49f-positive populations in undifferentiated MSC cultures. FTM: first trimester HUCPVC; term: term HUCPVC; BMSC: human bone marrow mesenchymal stem cell. Bar = 200 *μ*m. ^*∗*^ *p* ≤ 0.01.

Cardiomyogenic differentiation of MSCs (FTM HUCPVC, term HUCPVC, and BMSC) was induced by plating single cell suspensions of undifferentiated MSCs (henceforth direct cocultures) or MSC aggregates (aggregate cocultures) on a monolayer of primary rat cardiomyocytes. The distribution and morphologies of all three MSC types were similar at day 3 in direct cocultures (Figure 1(a)). However, FTM HUCPVCs and term HUCPVCs formed bigger aggregates (*d* = 300–500 *μ*m) than BMSCs (*d* < 200 *μ*m) (Figure 1(c)). We investigated whether CD49f (integrin subunit *α*6) [53, 54] expression differed in undifferentiated MSCs as a mechanism for differences in aggregate formation potential. FC analysis showed that 96% ± 3% of FTM HUCPVCs, 89% ± 6% of term HUCPVCs, and 53% ± 7% BMSCs express CD49f in culture (Figure 1(d)).

Seven days after coculture, FC analysis using anti-TRA-1-85 antibody identified low TRA-1-85-positive (TRA-1-85^low^) cells, distinguishable from both TRA-1-85-negative (rat) and high TRA-1-85-positive (human) cell populations (Supplementary Figure 1A, gate L). ICC for human nuclear antigen (HuNu, Supplementary Figure 1B) showed that TRA-1-85^high^ cells had single nucleus or multiple HuNu positive nuclei, while over 70% of TRA-1-85^low^ cells had several nuclei with various ratios of HuNu positive and negative labeling (Supplementary Figure 1B). This phenomenon was observed in all 3 MSC types and suggests the occurrence of some cell fusion in cardiomyocyte cocultures.

MSCs from direct cocultures were counted after differentiation (day 7). Starting with equal amount of cells (10k cells on day 0), we found remarkable differences in cell count between MSC types at the time of extraction (day 7). MSC numbers increased in both FTM HUCPVC- and term HUCPVC-containing cocultures (27k ± 7.1k, 17k ± 9.9k, resp.) but decreased in BMSC cocultures (3.8k ± 1.7k) (*p* < 0.01) (Figure 1(b)) suggesting a higher survival and proliferation rate of PVCs in cocultures.

**Figure 2:**
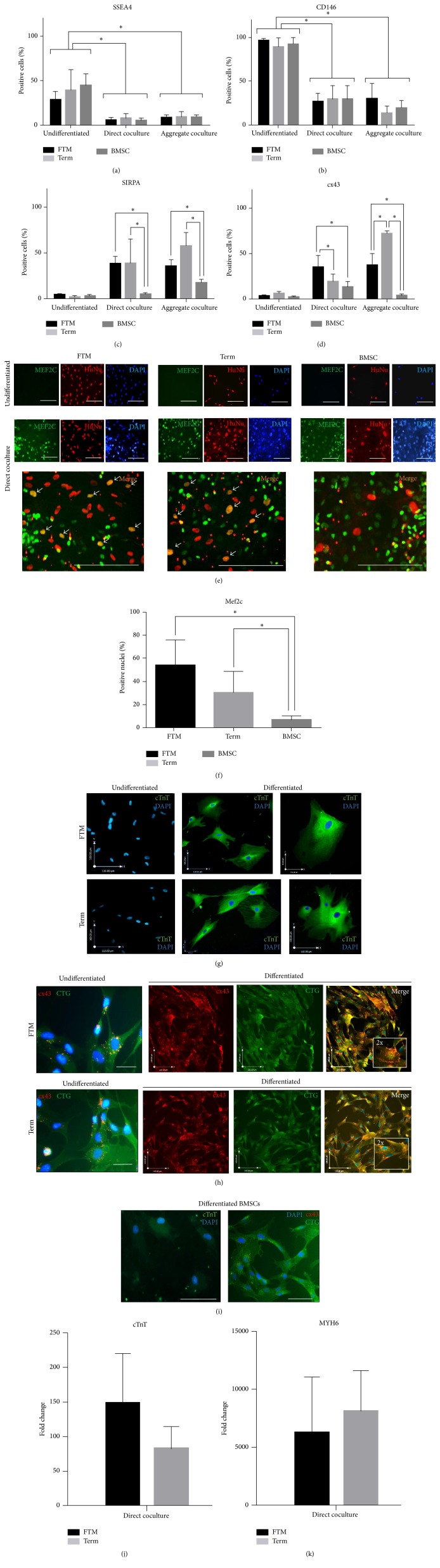
*In vitro* cardiomyogenic differentiation of human MSCs. (a–d) FC analysis of hMSCs. Undifferentiated MSC markers SSEA4 (a) and CD146 (b), cardiomyocyte marker SIRPA (c), and gap junction protein connexin 43 (cx43, (d)) levels expressed as % of overall human cell counts in undifferentiated and differentiated (direct coculture, aggregate coculture) first trimester PVC (FTM), term HUCPVC (term), and bone marrow (BMSC). *∗* indicates statistically significant difference (*p* < 0.01, *n*_FTM_ = 9, *n*_term_ = 9, *n*_BMSC_ = 6). (e) Confocal microscopy images of Mef2c (green) and HuNu (red) immunostaining in FTM and term HUCPVCs and bone marrow MSC containing rat primary cardiomyocyte cultures in comparison with undifferentiated MSCs. Blue: Hoechst (DAPI) Arrows mark double positive nuclei. Bar = 100 *μ*m. (f) Quantification of Mef2c and HuNu double positive nuclei in direct cocultures, expressed as % of HuNu positive (overall hMSC) counts. 50 independent, random fields of sight assessed per MSC type. ^*∗*^ *p* < 0.01. (g–i) Confocal microscopy images of TRA-1-85^high^ FTM and term HUCPVCs sorted from direct cocultures compared with undifferentiated cultures. Connexin 43 ((h, i), red) and cardiac troponin T ((g, i), green) stainings. For connexin 43, cells were counterstained with fixable dye (CellTracker Green). (h) Inner box: 2x magnification of representative fields showing intracellular distribution of cx43 positive puncta. Scale bars (e, i): 100 *μ*m; (g, h): *x* = 54 *μ*m and *y* = 48 *μ*m and *x* = 100 *μ*m and *y* = 110 *μ*m. Blue: Hoechst (DAPI filter). (j, k) Quantitative PCR analysis of cardiac troponin T (cTnT, (j)) and heavy chain cardiac myosin (MYH6, (k)) in TRA-1-85^high^ FTM and term HUCPVCs sorted from direct cocultures. Values expressed as fold change in comparison to undifferentiated cells. *n* = 4.

We analysed TRA-1-85^high^ cells for the expression of multipotent stromal cell marker SSEA4 [55], pericyte, and adult stem cell marker CD146 [56], as well as cardiomyocyte markers SIRPA and CX43 using FC. The majority of TRA-1-85^high^ MSCs (FTM, term, and BMSC) significantly downregulated SSEA4 expression in both direct coculture and aggregate coculture when compared to undifferentiated cells (Figure 2(a), *p* < 0.01). The proportion of CD146-positive cells significantly decreased during both direct coculture and aggregate coculture differentiations (Figure 2(b), *p* < 0.01). All 3 undifferentiated MSC types were found to be ≥90% CD146-positive and this significantly (*p* < 0.01) decreased in direct cocultures (26.9% ± 9.3%, 29.6% ± 15%, 27.6% ± 14%, resp.). The percentage of CD146 +ve cells further decreased in term HUCPVC and BMSC aggregate cocultures (13.6% ± 8% and 19.5% ± 8.2%, resp.), but not in FTM HUCPVC cocultures (30.4% ± 16.9%) (Figure 2(b)). Cardiomyocyte-associated marker SIRPA was upregulated in FTM HUCPVCs (35.6% ± 6.9%) and term HUCPVCs (57.9% ± 14.7%), but not in BMSCs (5.3% ± 2.6%) (Figure 2(c)). Significantly more FTM (35% ± 13%) compared to term (19.2% ± 7.8%) and BMSC (13.2% ± 5.8%) upregulated connexin 43 (cx43) (*p* < 0.01) (Figure 2(c)). Aggregate cocultures further increased SIRPA levels in term HUCPVCs (57.9% ± 14.7%) to significantly higher levels (*p* < 0.01) when compared to FTM (35.6% ± 6.9%) and BMSC (17.6% ± 3.4%). Concurrently, cx43 positivity of term HUCPVCs became significantly higher (72.3% ± 2.6%) than either FTM (37.2% ± 13%) or BMSC (3.8% ± 1.9%) in this assay (Figure 2(d)).

The percentage of Mef2c and HuNu double positive nuclei (Figure 2(e)) was significantly lower in BMSC cocultures (7% ± 3.2%) compared to FTM and term cocultures (54% ± 22.1%, 30.4% ± 18.6%, resp., *p* < 0.01Figure 2(f)). TRA-1-85-positive cells were sorted (Supplementary Figure 1A) and replated without primary cardiomyocytes to assess cardiomyocyte markers troponin T and gap junction protein cx43 (Figures 2(g)–2(i)). Differentiated FTM and term HUCPVCs showed intense cTnT staining with a distribution consistent with the cytoskeleton (Figure 2(g)). Although both FTM and term HUCPVCs upregulated cx43 during differentiation (Figure 2(h)), cx43 positive puncta predominantly localised in the plasma membrane of FTM cells as opposed to the cytoplasmic distribution observed in term cells. Fluorescent signal corresponding to cTnT and cx43 in differentiated BMSCs was low or undetectable (Figure 2(i)).

### 3.2. HUCPVCs Upregulate Cardiomyocyte-Specific Genes during* In Vitro* Differentiation

After coculture, the expression of human cTNT and MY6H genes in TRA-1-85^high^-sorted cells was analysed by qPCR. No expression was detected in undifferentiated cells. cTnT mRNA (Figure 2(j)) was upregulated in both FTM (148 ± 71-fold) and term HUCPVCs (81.7 ± 32-fold) following differentiation. MYH6 mRNA expression levels (Figure 2(k)) also greatly increased for both FTM (6200 ± 4800-fold) and term HUCPVCs (8100 ± 3500-fold) during differentiation. Human iPSC-derived cardiomyocytes (ReproCardio*™*) were used as a positive control and rat cardiomyocytes as negative control for these experiments.

### 3.3. Differentiating FTM HUCPVC Aggregates Spontaneously Contract on Rat Cardiomyocyte Feeder Layers

While no contracting term HUCPVC or BMSC aggregates were observed, 3 out of 5 FTM lines consistently produced aggregates that exhibited spontaneous pulsation after 5 days in coculture with primary cardiomyocytes (Videos 1, 2, and 3). Contracting cells were observed first in the inner parts of the aggregates as revealed by using prestained FTM HUCPVCs (CellTracker Green) (Video 1). Over 7 days the entire aggregate became motile (Videos 2–5) and preserved its activity even after the rat cardiomyocyte feeder layer stopped displaying physical activity (Video 4, Video 5). Furthermore, FTM HUCPVC aggregates sharing a common cardiomyocyte feeder layer exhibited synchronous contractions (Video 4, Video 5).

### 3.4. Human Umbilical Cord Perivascular Cells Show Decreased Immunogenicity When Compared to Bone Marrow-Derived MSCs

**Figure 3:**
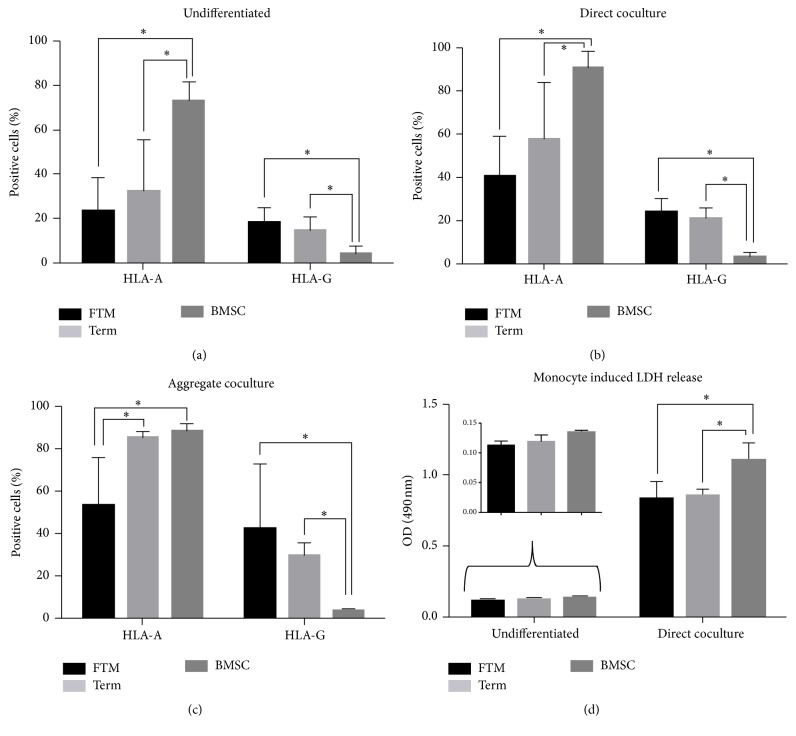
Immunological characteristics of hMSCs before and after cardiac differentiation* in vitro*. (a–c) FC analysis of HLA-A and HLA-G expression in (a) undifferentiated, (b) direct coculture, and (c) aggregate coculture differentiated FTM and term HUCPVCs and bone marrow MSCs (BMSCs). Values expressed as % of overall human cell number in corresponding cultures. (d) Colorimetric analysis of human leukocyte induced LDH release after 72 h of incubation with FTM and term HUCPVCs and BMSCs sorted from cardiomyocyte cultures (direct cocultures) compared to undifferentiated MSC cultures. Values expressed as optical density (OD) of LDH converted substrate detected at 490 nm. Inner graph shows undifferentiated MSC values (*y*-axis 3x of main graph). Asterisk indicates statistically significant difference (*p* < 0.01, *n*_FTM_ = 9, *n*_term_ = 9, *n*_BMSC_ = 6).

In undifferentiated cultures, HLA-A levels were significantly lower for FTM (23.5% ± 15.2%) and term HUCPVCs (32.3% ± 23%) when compared to BMSCs (70.3% ± 8.1%, *p* < 0.01) (Figure 3(a)). The proportion of immune privilege-associated HLA-G positivity was significantly higher in FTM (18.2% ± 6.4%) and term HUCPVCs (14.7% ± 6%) cells when compared to BMSCs (4.2% ± 2.8) (*p* < 0.01) (Figure 3(b)). Cardiomyogenic differentiation in cocultures led to upregulation of HLA-A in all MSC types, but HLA-A expression remained significantly lower for FTM (40.8% ± 18.2%) and term HUCPVCs (57.8% ± 26.1%) in comparison to BMSCs (90.7% ± 7.8%) (*p* < 0.01) (Figure 3(a)). In aggregate cocultures, proportions of HLA-A-positive cells increased in term HUCPVCs (85.4% ± 3%) but remained significantly lower in FTM HUCPVCs (53.6% ± 22.8%) (*p* < 0.01). HLA-G levels remained significantly higher in both direct cocultures (FTM: 23.8% ± 6.2%, term: 21% ± 4.7%) and aggregate cocultures (FTM 42.8 ± 30.4%, term 29.6% ± 6%) when compared to BMSCs (3.2% ± 1.7% and 3.7% ± 0.7%, resp.) (*p* < 0.01) (Figure 3(c)).

MSC differentiation led to an increased sensitivity to lymphocyte-mediated cytotoxicity, with FTM and term HUCPVCs being significantly less susceptible towards cellular cytotoxicity than BMSCs (Figure 3(d), inner panel).

### 3.5. FTM HUCPVCs Express Alpha-Sarcomeric Actinin after One Week of Coculture with Primary Rat Cardiomyocytes

**Figure 4:**
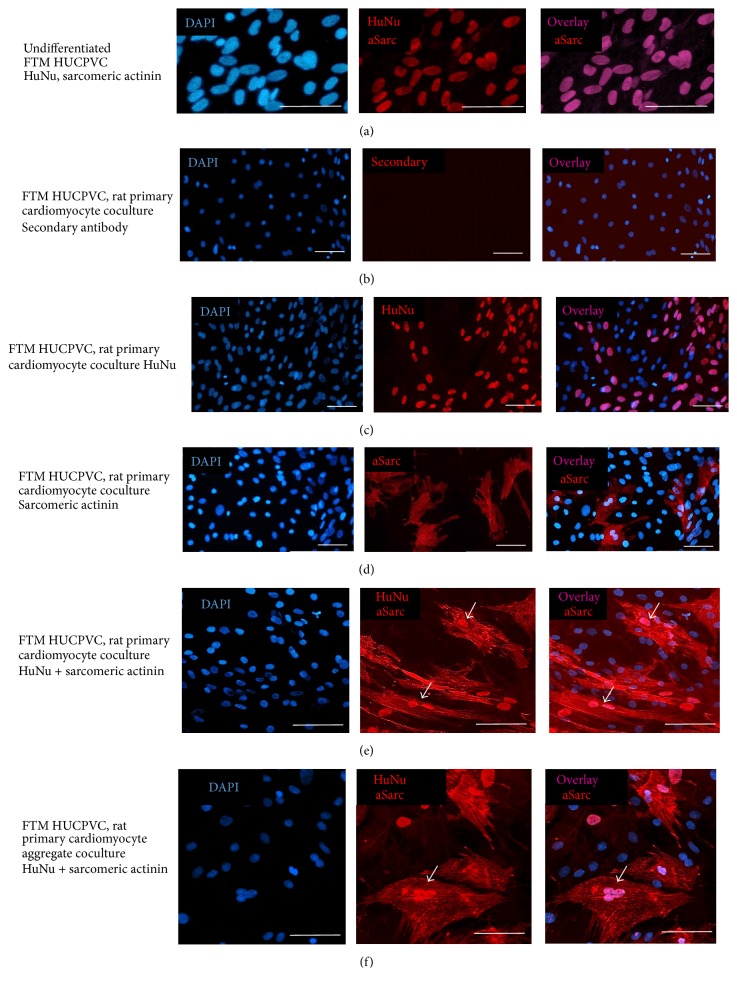
Alpha-sarcomeric actinin in FTM HUCPVCs after coculture differentiation. (a) Undifferentiated FTM HUCPVCs costained with human nuclear antigen- (HuNu-) specific and alpha-sarcomeric actinin- (aSarc-) specific antibodies. (b–d) Rat primary cardiomyocyte, FTM HUCPVC cocultures stained with secondary antibody only (b), human nuclear antigen- (HuNu-) specific (c) or alpha-sarcomeric actinin- (aSarc-) specific (d) antibody. (e, f) Rat primary cardiomyocyte, FTM HUCPVC cocultures costained with human nuclear antigen- (HuNu-) specific and alpha-sarcomeric actinin- (aSarc-) specific antibodies. DAPI: nuclear stain. Bar = 100 *μ*m.

The emergence of contracting cells from FTM HUCPVCs suggested the expression of alpha-sarcomeric actinin after differentiation. We performed immunocytochemistry on undifferentiated and differentiated FTM HUCPVCs (Figure 4) in order to detect alpha-sarcomeric actinin (aSarc) in cells with human nuclear antigene (HuNu) positive nuclei. While undifferentiated FTM HUCPVCs did not show alpha-sarcomeric actinin expression (Figure 4(a)), FTM HUCPVCs in direct cocultures with primary rat cardiomyocytes for 1 week (Figure 4(e)) contained alpha-sarcomeric actinin expressing cells and human nuclear antigen-positive nuclei (Figure 4(e) arrows). Aggregate cocultures of FTM HUCPVCs and primary rat cardiomyocytes were lifted after one week and were replated for alpha-sarcomeric actinin and human nuclear antigen immunostaining (Figure 4(f)). Microscopy revealed cells from aggregate cocultures with strong positive staining for alpha-sarcomeric actinin (aSarc) that contained exclusively human nuclear antigen- (HuNu-) positive nuclei (Figure 4(e), arrow).

## 4. Discussion

Our study supports 3 main novel conclusions as follows: (1) Young human umbilical cord perivascular cells (HUCPVCs) possess increased* in vitro* cardiomyogenic potential when compared to BMSCs. (2) While similar epigenetic changes and upregulation of mature cardiomyocyte markers were observed in both FTM and term HUCPVC cocultures with cardiomyocytes, FTM HUCPVCs exclusively were able to generate contracting cells in aggregate cocultures. (3) Differentiated FTM and term HUCPVCs have significantly lower immunogenicity than BMSCs* in vitro*.

To our knowledge, FTM HUCPVCs are the first described source of human MSCs with the ability to differentiate into functional (contracting) cardiomyocyte-like cell* in vitro*. In addition to a steep elevation of cardiac differentiation marker expression (Mef2c, SIRPA, cTnT, MYH6, and cx43) observed during HUCPVC differentiation in primary cardiomyocyte cocultures, we found that FTM HUCPVCs from aggregate cocultures developed contracting cells within 1 week and express alpha-sarcomeric actinin.

The generation of contracting cardiomyocyte-like cells in aggregate cocultures suggests that the aggregation-induced hypoxic micromilieu might play an important role in initiating differentiation of MSCs. Although aggregate generation and hypoxic preconditioning are established strategies for human ESC-based cardiac differentiation [16, 57, 58] and the control of oxygen tension during the differentiation process is an essential feature of cardiac tissue engineering [19], the relevance of such approaches for human MSC applications has not been thoroughly studied. This finding suggests that strategies enhancing aggregate formation could increase the efficiency of HUCPVC-based differentiation for optimized tissue engineering applications. To elucidate the underlying cause of the observed difference in aggregate formation potential between BMSCs and HUCPVCs, we examined the expression of CD49f (integrin alpha 6) in FTM and term HUCPVCs in comparison with BMSCs. CD49f is in direct regulatory connection with the pluripotency markers OCT4 and SOX2, denoting cells with higher stemness [53], and is shown to be crucial determinant of sphere formation potential of MSCs [53]. We previously demonstrated that FTM HUCPVCs have increased OCT4A expression when compared to term counterparts or BMSCs [46]. CD49f-positive MSCs were also reported by others to have increased cardiac regenerative capacity in a post-MI mouse model [54]. Here, we demonstrated that CD49f expression levels are increased in HUCPVCs relative to BMSCs and that this correlates with their aggregate formation capacity and cardiomyogenic differentiation potential.

The immunomodulatory effect of administered cells, through both secreted factors and expression of cell surface molecules, is an important determinant of* in vivo* engraftment. Based on their source and age, MSCs vary in expression of CAMs, HLA molecules, and also T cell activation and response [59]. While HLA-A levels determine susceptibility towards cytotoxic T cells, the immunomodulatory protein HLA-G attenuates the response of the innate immune system [60]. Representation of these antigens on engrafted cells could define a host's cellular immune response after allogeneic transplantation. Our findings are in agreement with previous reports that mesenchymal stromal cells of umbilical cord origin display lower HLA-A and higher HLA-G expression levels when compared to adult or other extraembryonic tissue originated MSCs [61]. Immunogenic properties of BMSCs have been shown to change during differentiation and lead to rejection in allogeneic models [62]. Undifferentiated FTM and term HUCPVCs exhibit low HLA-A levels but express HLA-G that provides protection for fetal tissues* in utero*. Although term HUCPVCs upregulated HLA-A during aggregate-based cocultures, FTM HUCPVCs maintained low HLA-A expression, and both HUCPVC types sustained or upregulated HLA-G in the course of differentiation* in vitro*. Under the same experimental conditions, BMSCs exhibited the highest HLA-A expression and negligible HLA-G positivity. Furthermore a functional assay measuring T cell reaction (LDH release) showed a significantly higher susceptibility of differentiated BMSCs towards T cell mediated cytotoxicity compared to HUCPVCs.

Based on our results we propose that FTM HUCPVCs have superior cardiomyogenic differentiation potential and favorable immunogenic properties when compared to human MSCs derived from older tissue sources that make them highly promising candidates for cardiac tissue engineering and* in vivo* cardiovascular regenerative applications.

## Supplementary Material

Supplementary Figure 1: (A) Flow cytometry gating strategies for selecting human cells in human MSC, rat cardiomyocyte co-cultures. APC and FITC conjugated anti-human cell surface marker TRA-1-85 applied on human MSC free rat cell cultures (upper row) and human, rat co-cultures (lower row). Cell populations with high (H) or low (L) positivity for TRA-1-85 were identified.
(B-C) Immunocytochemistry on cells sorted from rat, human co-cultures using anti-TRA-1-85 antibodies (A). Human nuclear antigen (HuNu, red) was applied to reveal nuclei of human origin. High TRA-1-85+ cells displayed exclusively HuNu positive nuclei (B), low TRA-1-85+ cells displayed both HuNu positive and negative nuclei.
Supplementary Figure 2: (A-C) Validation of flow cytometry antibodies used for human cardiac marker quantification. (A) anti-human SIRPA (PE) antibody applied on rat primary cardiomyocyte culture. (B-C) anti-human connexin 43 (cx43, APC) and anti-human specific marker TRA-1-85 (FITC) applied on rat primary cardiomyocyte culture. Positive cell populations were quantified by depicting APC and FITC signals individually (B) or in combination (C). TRA-1-85 negative, cx43 positive cell population was identified within the co-culture (C, box).
(D) Validation of human cTnT and human MYH6 specific qPCR primers on human heart cDNA and rat primary cardiomyocyte cDNA samples. Amplification and melting curves (upper row) show purity of the reactions. Table (lower row) shows Ct values with each primer pair applied on each sample.
