# Supplementary material for: In Vitro Differentiation of First Trimester Human Umbilical Cord Perivascular Cells into Contracting Cardiomyocyte-Like Cells
Source: Stem Cells Int. 2016 Mar 30;2016:7513252. doi: 10.1155/2016/7513252 (PMC4829731; doi:10.1155/2016/7513252)
Supplement: Supplementary file 1 — Supplementary Figure 1: (A) Flow cytometry gating strategies for selecting human cells in human MSC, rat cardiomyocyte co-cultures. APC and FITC conjugated anti-human cell surface marker TRA-1-85 applied on human MSC free rat cell cultures (upper row) and human, rat co-cultures (lower row). Cell populations with high (H) or low (L) positivity for TRA-1-85 were identified. (B-C) Immunocytochemistry on cells sorted from rat, human co-cultures using anti-TRA-1-85 antibodies (A). Human nuclear antigen (HuNu, red) was applied to reveal nuclei of human origin. High TRA-1-85+ cells displayed exclusively HuNu positive nuclei (B), low TRA-1-85+ cells displayed both HuNu positive and negative nuclei. Supplementary Figure 2: (A-C) Validation of flow cytometry antibodies used for human cardiac marker quantification. (A) anti-human SIRPA (PE) antibody applied on rat primary cardiomyocyte culture. (B-C) anti-human connexin 43 (cx43, APC) and anti-human specific marker TRA-1-85 (FITC) applied on rat primary cardiomyocyte culture. Positive cell populations were quantified by depicting APC and FITC signals individually (B) or in combination (C). TRA-1-85 negative, cx43 positive cell population was identified within the co-culture (C, box). (D) Validation of human cTnT and human MYH6 specific qPCR primers on human heart cDNA and rat primary cardiomyocyte cDNA samples. Amplification and melting curves (upper row) show purity of the reactions. Table (lower row) shows Ct values with each primer pair applied on each sample. [file 7513252.f1.zip › SuppFigure2_120216.pptx]

## Slide 1
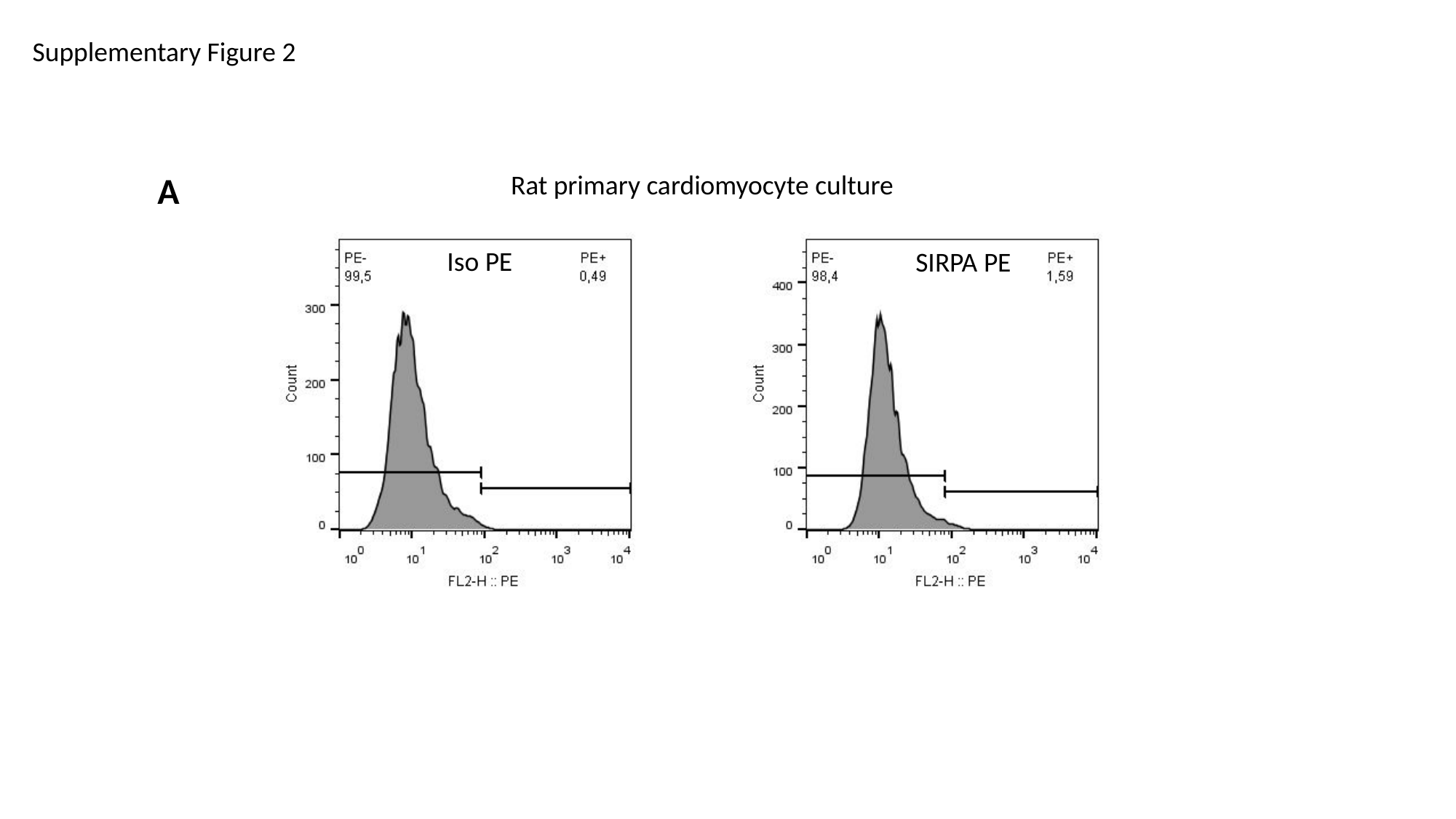

Supplementary Figure 2
A
Rat primary cardiomyocyte culture
Iso PE
SIRPA PE

## Slide 2
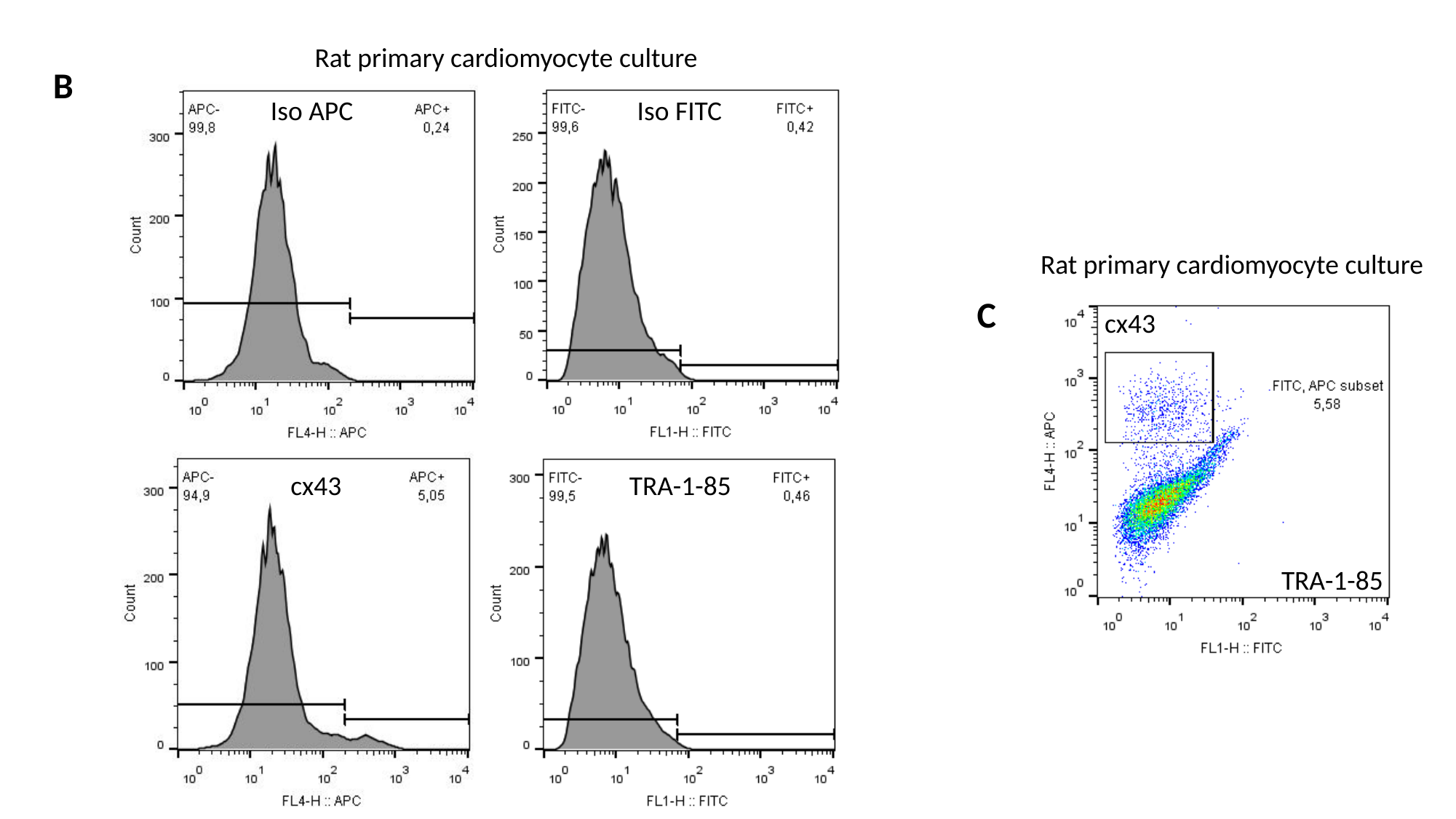

Rat primary cardiomyocyte culture
B
Iso APC
Iso FITC
Rat primary cardiomyocyte culture
C
cx43
cx43
TRA-1-85
TRA-1-85

## Slide 3
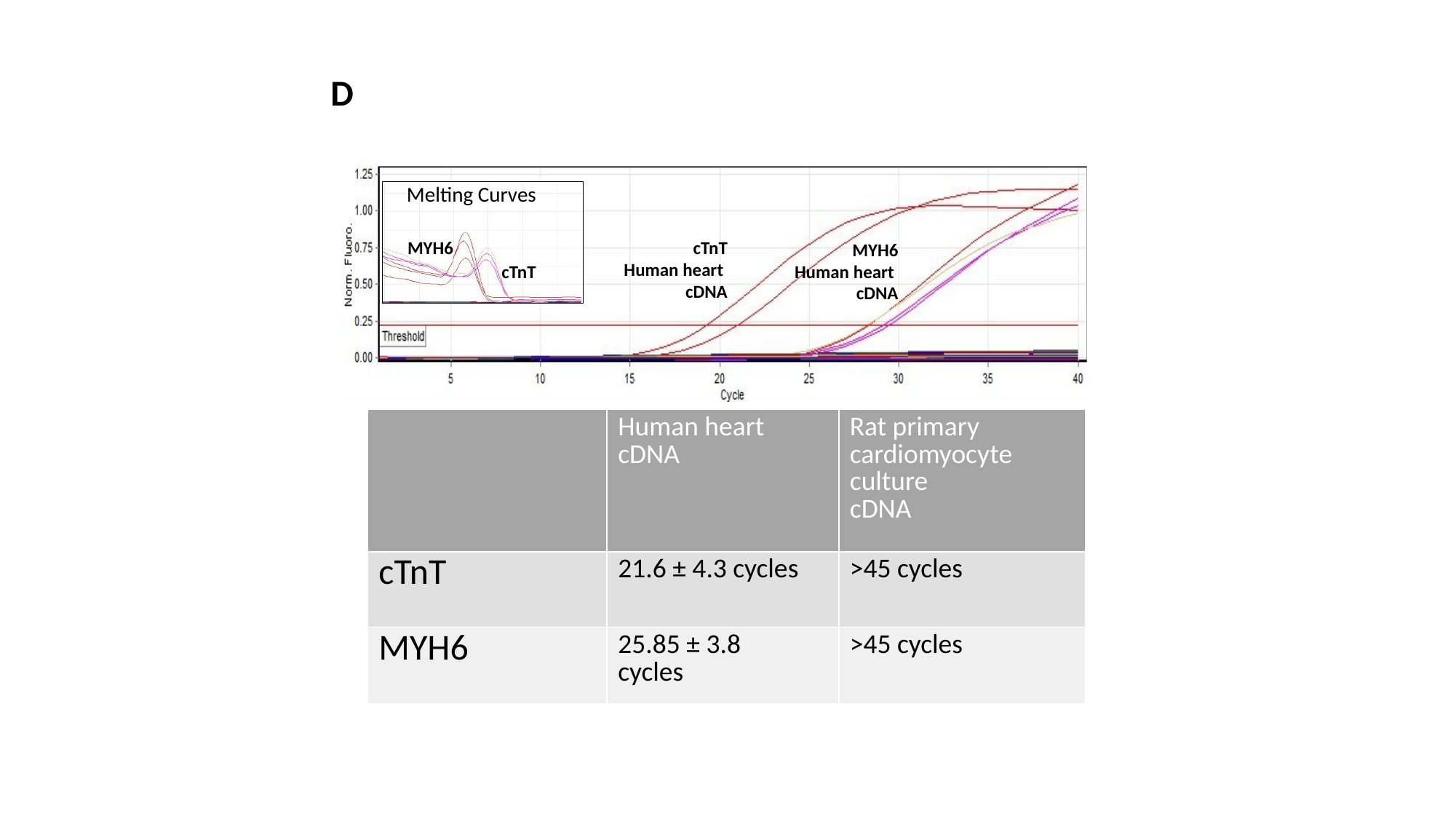

D
Melting Curves
MYH6
cTnT
Human heart
cDNA
MYH6
Human heart
cDNA
cTnT
| | Human heart cDNA | Rat primary cardiomyocyte culture cDNA |
| --- | --- | --- |
| cTnT | 21.6 ± 4.3 cycles | >45 cycles |
| MYH6 | 25.85 ± 3.8 cycles | >45 cycles |
